# COVID-19 pandemic influence on perceived exposure to chemical substances in Latvia: data from a focus group discussion and the HBM4EU citizen survey

**DOI:** 10.3389/fpubh.2024.1382368

**Published:** 2024-05-23

**Authors:** Lāsma Akūlova, Linda Paegle, Inese Mārtiņsone, Ivars Vanadziņš, Lisbeth E. Knudsen, Linda Matisāne

**Affiliations:** ^1^Laboratory of Hygiene and Occupational Diseases, Institute of Occupational Safety and Environmental Health, Rīga Stradiņš University, Riga, Latvia; ^2^Institute of Occupational Safety and Environmental Health, Rīga Stradiņš University, Riga, Latvia; ^3^Department of Public Health, University of Copenhagen, Copenhagen, Denmark

**Keywords:** COVID-19, exposure, chemical substances, focus group discussion, disinfectants

## Abstract

**Introduction:**

The COVID-19 pandemic has globally influenced the exposure of populations to chemical substances through various channels. This study aims to evaluate the tendencies of the use of chemical products in Latvia amidst the pandemic. Answers from 597 respondents (26.6% male, 73.4% female, mean age 46.0 ± 12.2) which were gathered as part of the HBM4EU (Human Biomonitoring Initiative) citizen survey and 8 focus group participants were used.

**Methods:**

The study utilized data from the HBM4EU citizen survey and conducted focus group discussions to understand the impact of the COVID-19 pandemic on chemical product usage in Latvia. Survey responses were analyzed to identify changes in exposure to chemicals, particularly in relation to disinfection agents and household products.

**Results:**

More than two-thirds of survey participants reported increased exposure to chemicals during the COVID-19 pandemic, mainly related to the use of disinfection agents and household products. About 2-in-5 (39.8%) of survey respondents considered that the COVID-19 pandemic has increased their interest in exposure to chemicals. The excessive use of disinfectant products is the main concern of citizens (mentioned by 66.7%, n = 389). Also, two focus group participants noted that the use of disinfectant products is too widespread and should be minimized.

**Discussion:**

The findings suggest that the COVID-19 pandemic has not only increased the use of chemical products in Latvia but also promoted an interest in safe and healthy use of chemicals which could be useful to raise the awareness of the general public.

## 1 Introduction

Through everyday tasks such as commuting and traveling, cooking, cleaning, doing laundry, etc., humans act as polluters of the water, soil and air. However, the coronavirus COVID-19 disease (COVID-19) pandemic and strong measures that were taken to limit the spread of the virus altered our daily habits (1–3). The described environmental benefits of the COVID-19 pandemic lockdowns include reduced noise pollution and less environmental waste due to limited tourism (1, 2), however, these changes have reverted once the restrictions lessened (4). This means that the effects of the pandemic continue to impact the environment on top of the already existing problems – additional waste (especially medical-related) and reduced rates of recycling (2).

The coronavirus disease of 2019 has been a persistent challenge to the global healthcare system since 2019. The most common symptoms of SARS-CoV-2 infection are cough, fever, chills, difficulty in breathing and pain (overall body and headache). Due to genetic mutations, at least 10 significant variants of SARS-CoV-2 have emerged, challenging innovations in vaccine development (5). Globally, the fight to limit the spread of COVID-19 has been mostly based upon immunization, massive screening, social distancing, proper ventilation, frequent window opening and other measures such as the use of disinfectants and personal protective equipment (PPE) – face masks, respirators, gloves, etc. (2, 4, 6, 7). In addition, during the lockdowns time spent indoors increased, and this has drawn focus on human exposure to indoor air pollution (7).

According to an assessment done by the United Nations Development Programme, in Asia, hazardous healthcare waste generated from COVID-19 testing, vaccination and PPE increased 10 times in volume during the peak of the pandemic compared to the time before resulting in 3.4 kg extra waste per bed per day (8). By assuming that all single-use PPEs will end up as waste, the distributed amount equals the amount of waste (8). It is estimated that since the outbreak 1.6 million tonnes of PPE and plastic waste is generated per day and approximately 3.4 billion single-use facemasks and shields are disposed daily worldwide. Estimated data shows that 153,623 tonnes of plastic waste per day are generated in Europe (9). The pandemic also influenced businesses and manufacturers – restaurants offered take-away options or deliveries only and shop owners relied on online shopping to keep their businesses open. While lockdowns negatively impacted restaurant traffic, delivery firms reported a 50% increase in breakfast orders and 80% in lunch orders in April, May and June 2020, compared to a similar period in 2019 (10). This resulted in increased usage of single-use plastics, packaging and filling materials for safe transportation of goods (11).

Since human-to-human transmission via respiratory droplets and contact with aerosol-infected surfaces are the major ways of transmitting this virus (12), in order to limit the spread of the SARS-CoV-2 coronavirus, the use of face masks during the COVID-19 pandemic was mandatory in most public places. Additional waste was not the only problem resulting from the use of face masks. By using them, people were exposed to several chemical compounds as face masks are usually made of polymers (mostly polypropylene) and contain many other chemicals which are added during the manufacturing process, e.g., phthalates, formaldehyde and others (13, 14).

Although the SARS-CoV-2 coronavirus is not resistant to environmental exposure, it can survive hours, even days on surfaces, depending on the material (12, 15). This has been combated by using disinfectants and cleaning products. In March 2020, the European Centre for Disease Prevention and Control issued a technical report on the disinfection of healthcare and non-healthcare facilities. It suggested that after the presence of a suspected or confirmed case of COVID-19, all non-healthcare premises should be regularly ventilated and surfaces cleaned using a neutral detergent, followed by disinfection. It was also advised that disposable, single-use equipment be used to minimize the risk of infection, however, this also increased the amount of waste generated due to COVID-19. During the COVID-19 pandemic, it was requested that frequently touched surfaces be wiped at least daily in all types of premises. Neutral detergents had to be used to clean the surfaces and the cleaning materials had to be properly cleaned after each cleaning session. Frequent hand washing and disinfection were also suggested as helpful in maintaining personal health and hygiene as well as limiting the possibility of infection in all premises (16).

Lockdowns and restrictions caused behavioral changes. Studies have indicated an increase in the use of household chemical products during the COVID-19 pandemic. In comparison with the pre-pandemic period, the frequency of cleaning had increased by 69.3% and the amount of cleaning product usage by 74.2%. Since household chemicals contain several chemical compounds, the increased usage of these products caused indoor air pollution and posed a risk to human health. Together with lockdowns and social isolation, even curfew hours, the amount of time spent at home increased as well and the quality of indoor air became even more important than pre-pandemic (17). Combined effects of chemical exposure and the complexity of this issue (compounds, concentrations, usage suggestions, etc.) could lead to misconceptions and reduced interest in this topic, resulting in low awareness of chemical exposure in everyday life. The lack of information and insufficient environmental education determined citizens’ perceptions and actions on chemical product usage (18).

At the same time, it is important to illustrate the situation with the COVID-19 pandemic in Latvia before and during the data collection related to this research paper. During the first year of the pandemic (before the focus group discussions on chemical exposure were carried out), Latvia was not among the countries most affected by the pandemic in terms of cases. In Latvia, from the 3rd of January 2020 to the 19th of March 2024, there were 997,701 confirmed cases of COVID-19 with 7,465 deaths (19). The majority of confirmed cases were registered on the 19th of February 2022–11,992 cases. Most single-day death cases were recorded on the 17th of November 2021 (*n* = 79) (20). At the very start of the pandemic (March 2020), the growing number of infections forced the Latvian government to take action, resulting in the announcement of an emergency state due to COVID-19. These measures included closing schools and promoting remote work (where possible), cancelation of public events, restrictions on gatherings, etc. (21). Most of the restrictions were removed in April 2023 (22).

Although there are several studies on the citizens’ perceptions of exposure to chemical substances and the influence of the COVID-19 pandemic globally, currently, there are no published data on the situation in Latvia resulting in the research gap. It has also been stressed by the European research community that further research on the impact of the COVID-19 pandemic on the exposure of citizens to chemicals should be done (23). Therefore, this paper provides an additional basis for further policy implications in the protection of European citizens and better preparedness for the management of possible pandemics in the future. This article will give a broader insight into the influence of the COVID-19 pandemic on human health and the usage of chemical products in a high-income European Union country. This paper aims to discuss the impact of the COVID-19 pandemic on the usage, attitudes and citizen exposure to chemical substances in Latvia and will add valuable insight for national as well as international researchers in this field.

## 2 Materials and methods

Quantitative and qualitative research methods were used to gather information: a web-based questionnaire was used for citizen survey in Latvia and a focus group discussion was organized to gather in-depth information. This research was done using national data gathered as part of the European Human Biomonitoring Initiative (HBM4EU) which was a project that studied human exposure to various chemicals and their long-term effects on health. HBM4EU was a cooperation initiative of 30 European countries and the European Environment Agency and the European Commission. The results provided an opportunity for policy change in the use of chemicals (24).

The study in Latvia was conducted by the Institute of Occupational Safety and Environmental Health which is a unit at the Rīga Stradiņš University (hereinafter IOSEH), therefore, it was approved by the Ethics Committee of Rīga Stradiņš University (protocol No. 22–2/250/2021, the 14^th^ of April 2021). This research was funded by the European Union’s Horizon 2020 research and innovation program under grant agreement No 733032.

### 2.1 The citizen survey

#### 2.1.1 Recruitment and data collection

The citizen survey was translated and tested in 20 national languages of the countries participating in the HBM4EU initiative. A similar questionnaire in all participating countries was used and it was based on Microsoft Forms as a tool to gather answers from respondents. The citizen survey applied a non-probability sampling method. Survey participants were recruited using a snowball sampling method, social media advertisements as well as direct emails to share the web link of the questionnaire in Latvian. The researchers from all countries organizing citizen surveys agreed not to use inclusion and exclusion criteria, therefore no filter questions were used. Every single person having access to the internet and having sufficient digital skills was able to fill in the questionnaire. The link to the questionnaire in Latvian was active between the 15th of September 2020 and the 28^th^ of February 2021 and it took about 15 min to complete the survey. These answers characterize the opinion of the citizens during the 1st wave of the COVID-19 pandemic.

The promotion and distribution of the survey link were done in several phases. The first phase took place in mid-September 2020, primarily through various social networking platforms. The national occupational health and safety portal,^1^ as well as the Twitter account of the IOSEH and the Facebook account of the cooperation partner – the State Labor Inspectorate were the main sources. The second phase of dissemination of the questionnaire was launched on the 23rd of November 2020, when additional partners were invited to distribute the link among their members (the Free Trade Union Confederation and the Employers’ Confederation of Latvia). Direct personal emails were also sent to the professional contacts of the IOSEH.

Prior to the survey, estimates were calculated to reach the required population using a 5% margin error, 99% confidence interval, 50% response rate, and 2.368 million inhabitants in Latvia in the 2^nd^ quarter of 2020, resulting in 663 persons. Despite repeated recruitment efforts, only a total of 624 respondents completed the questionnaire. In the process of creating data weights, 27 respondents were excluded from further analysis as these respondents did not specify their gender and/or age, leaving a total of 597 participants for the analysis. Thus, the study group consisted of 73.4% (*n* = 438) women and 26.6% (*n* = 159) men. Most respondents were 35–44 years old (24.8%, *n* = 148) and 45–54 (28.3%, *n* = 169). More than 70% had obtained higher education (87.8%, *n* = 524) and 71.6% (*n* = 426) were employees. Additional information on the characteristics of the study group is given in Table 1.

**Table 1:** Distribution of the study sample, *n* (%).

**Total sample**	
**Gender**	
Female	438 (73.4%)
Male	159 (26.6%)
**Age**	
20–24 years	19 (3.2%)
25–34 years	98 (16.4%)
35–44 years	148 (24.8%)
45–54 years	169 (28.3%)
55–64 years	126 (21.1%)
>65 years	37 (6.2%)
**Highest level of education**	
Elementary school education	1 (0.2%)
Secondary school education	28 (4.7%)
Vocational secondary education	40 (7.3%)
Higher education	524 (87.8%)
**Current work situation**	
Self-employed	43 (7.2%)
Employee	426 (71.6%)
Employer	12 (2.0%)
Civil servant	72 (12.1%)
Pension/retirement	14 (2.3%)
In education/training	13 (2.2%)
Job seeker	8 (1.3%)
On maternity leave	6 (1.0%)
Disabled person	2 (0.3%)
**Size of the town**	
Less than 5.000 inhabitants	88 (14.7%)
5.001 to 20.000 inhabitants	161 (27.0%)
20.001 to 100.000 inhabitants	118 (19.8%)
100.001 to 500.000 inhabitants	27 (4.5%)
More than 500.000 inhabitants	167 (28.0%)
I do not know	36 (6.0%)

#### 2.1.2 Study variables

The questionnaire covered 21 questions (including 8 demographic characteristics), but this research explores two questions that are related to the influence of the COVID-19 pandemic: (1) on changes of interest regarding chemical products and (2) the perceived impact on health. The first question included a general assessment of the changes in the situation as a result of the pandemic and read: “Did the COVID-19 pandemic influence your interest in exposure to chemicals?.” The respondent had the opportunity to express his/her opinion by choosing one of the following answers: “Yes, markedly,” “Yes, slightly,” “No,” and “I do not know.” In the following question “In your opinion, do you feel that exposure to chemicals has changed during the COVID-19 pandemic?” the respondent was asked to clarify and specify the changes in exposure to all specified chemicals, which included an assessment of changes in the environment (natural and domestic), food and drink, various medicine, household, personal care products and, of course, disinfection agents and PPEs (all statements are given in Table 2). Three possible answers were provided per each source of chemicals: “Has increased,” “Did not change” and “Has decreased.”

**Table 2:** COVID-19 pandemic influence on respondent interest in exposure to chemicals.

		**15–24**	**25–34**	**35–44**	**45–54**	**55–64**	**65+**
Yes, markedly (9.3%, *n* = 55)	Man (9.1%, *n* = 25)	0 (0%)	8 (32.0%)	4 (16.0%)	1 (4.0%)	10 (40.0%)	2 (8.0%)
	Woman (9.4%, *n* = 30)	2 (6.7%)	0 (0%)	6 (20.0%)	5 (16.7%)	9 (30.0%)	8 (26.6%)
Yes, slightly (30.6%, *n* = 182)	Man (29.1%, *n* = 80)	0 (0%)	24 (30.0%)	16 (20.0%)	12 (15.0%)	11 (13.8%)	17 (21.2%)
	Woman (32.0%, *n* = 102)	7 (6.8%)	16 (15.5%)	14 (13.6%)	19 (18.4%)	23 (22.3%)	24 (23.4%)
No (56.2%, *n* = 334)	Man (57.4%, *n* = 158)	19 (11.9%)	24 (15.0%)	28 (17.5%)	36 (22.4%)	31 (19.4%)	22 (13.8%)
	Woman (55.2%, *n* = 176)	7 (4.0%)	33 (18.8%)	33 (18.8%)	29 (16.4%)	30 (17.0%)	44 (25.0%)
I do not know (3.9%, *n* = 23)	Man (4.4%, *n* = 12)	0 (0%)	0 (0%)	6 (50.0%)	4 (33.3%)	0 (0%)	2 (16.7%)
	Woman (3.4%, *n* = 11)	1 (9.1%)	3 (27.3%)	2 (18.2%)	3 (27.2%)	2 (18.2%)	0 (0%)

#### 2.1.3 Statistical analysis

Descriptive (mean, standard deviation) and frequency analysis were used to describe the data. The following age groups were used: 18–24, 25–34, 35–44, 45–54, 55–64 and + 65.

To compensate for the gender disproportion, data weights from the age and gender combinations of the Latvian population were created. For this, the public information from the Central Statistical Bureau of Latvia on the population of Latvia by gender and age (20–79 years) of 2020 was used. Since the Central Statistical Bureau of Latvia does not provide a breakdown of the level of education, using weights for education was impossible. Both results with and without weights (uncorrected survey data) are described in the section of results and available in tables in the same section.

Data analysis was performed with statistical software IBM SPSS, version 27 (IBM Corporation, Armonk, New York, NY, United States).

### 2.2 Focus group discussions

Between February 2018 and October 2020, a cross-sectional qualitative study - semi-structured focus group interviews were conducted in four European countries – Austria, Portugal, Ireland, and the United Kingdom (25). Later it was decided to organize additional focus group interviews to evaluate the perceptions and opinions in other countries in the European region where the attitudes toward chemical safety might differ. From October 2020 to August 2021, focus group interviews were carried out in seven countries: Cyprus, Denmark (two groups), the Netherlands (four groups), Hungary, North Macedonia, Israel, and Latvia (in August 2021), using a similar approach as for the first four discussions (25). For the last focus group discussions, the content was adjusted to the global changes related to the COVID-19 pandemic. For the purposes of this research, only the questions related to concerns of the COVID-19 pandemic on exposure to chemical substances were used.

#### 2.2.1 Recruitment and description of the study population

The participants for the focus groups were recruited using social media (Facebook, Twitter) posts and public announcements. Additionally, targeted boosting and a press release in a national occupational safety and health portal^2^ were published inviting people to participate. To obtain a heterogeneous group and equal representation of age, sex and education level, researchers invited the applicants to fill in an online form and to specify their age, sex, and education level. At no point in this study it was asked to declare if the person applying for participation in the focus group discussion had also participated in the citizen survey, therefore, the authors cannot exclude such a possibility. In total, 17 persons responded. After the evaluation which was done by the research team, 12 participants were individually contacted and 10 of them approved their participation. Prior to the attendance, an online informed consent form was filled in and gathered from the participants. Due to personal reasons, only eight participants were able to join the discussion. Out of all participants majority (six) were men. Four people were in the age group 30–44, three participants were aged 45–59 years and one person was in the 60–74 age group. Despite the efforts to gather a group of people from various educational backgrounds, most participants had obtained higher education (six held a university diploma), and the remaining two had finished secondary school/vocational training. The participants of the focus group were not specifically asked to introduce themselves to this topic prior to the discussion.

#### 2.2.2 Setting and framing of the discussion

To comply with the COVID-19 epidemiological restrictions, the focus group was organized in online settings, using the ZOOM platform. A standardized procedure - structured guidelines with questions that logically proceeded one after another was used for focus group discussions. An experienced moderator (I.V.) led the group. The moderator has general knowledge of this topic; however, he is not an expert in chemistry or environmental sciences. The discussion was technically supported by a note-taker (L.A.). After obtaining oral consent, the focus group was recorded and the record was used in transcription, avoiding incorrect information interpretation. All data protection rules were taken into account and the recording of the focus group is stored on the internal server of the institution.

The moderator started the discussion by introducing himself and shortly explaining the main idea of the focus group and establishing ground rules. Everyone was encouraged to speak freely stressing that each opinion is worthy and respectful. After the introduction, participants introduced themselves. Then the moderator carried on by asking different questions about participants’ perceptions and concerns regarding human biomonitoring and exposure to chemicals. The questions regarding the COVID-19 pandemic were asked more toward the end of the discussion, taking approximately 10 min (the total duration was 115 min) and the following question was asked: “The COVID-19 pandemic changed our lives significantly in the past year. As a result, our habits changed. What is your opinion – has it somehow influenced the use of chemical substances in everyday life? Which part of everyday life has changed the most? How much have these changes influenced our exposure to chemical substances?.” For the convenience of the moderator and participants, the questions were combined in a PowerPoint presentation and shared when the relevant question was discussed.

#### 2.2.3 Data analysis

The recorded focus group discussion was later transcribed and anonymized (L. P.). One day after the discussion, one of the participants sent in additional answers that were integrated into the suitable paragraphs and marked as “submitted later.” The participants were deidentified by an expert who was not involved in the transcription or analytical process (L. A.). Coding, careful systematic analysis and interpretation were performed by two experienced experts with different backgrounds (L. M., I. M.). Later on, conventional content analysis was performed.

Tentative categories were built based directly on the data, not on theoretical considerations. This allowed the researchers to carry out an unprejudiced assessment and was seen as the best possible approach. Simultaneously, subcategories were formed in the process of analysis while working with the transcription and were later refined, collapsed and merged into the final set of categories. Throughout the analysis of the transcript, the most relevant quotes were marked and examples of best describing the different answers and perceptions were used to demonstrate different opinions and experiences. To provide a wider perception of the group opinions, it was decided to reference the age, sex and education level of each of the participants.

## 3 Results

### 3.1 Results of the citizen survey

The citizen survey results showed that 56.2% (*n* = 335) of the respondents thought that the COVID-19 pandemic had not influenced their interest in exposure to chemicals. No significant and statistically reliable gender and age differences were observed among respondents, who gave a negative answer to this question (47.3% of men, *n* = 158 vs. 52.7% of women, *n* = 176) (for details see Table 2).

About 2-in-5 participants (39.8%, *n* = 237) responded that the COVID-19 pandemic has increased their interest in exposure to chemicals, including only 9.2% (*n* = 55) admitting that the interest has increased markedly. The majority of the respondents with increased interest have highlighted that the interest has been increased slightly (30.6% of all respondents, *n* = 182). However, among respondents who answered that the COVID-19 pandemic had increased their interest in exposure to chemicals, there were slightly more women than men. The difference between both genders was approximately equal among those with markedly increased interest (54.5% of women, *n* = 30 vs. 45.5% of men, *n* = 25) and those with slightly increased interest (56.0% of women, *n* = 102 vs. 44.0% of men, *n* = 80).

Respondents were also asked to evaluate the changes in exposure to chemicals from different exposure sources (Table 3). Most of the respondents (68.8%, *n* = 408) noted they felt their exposure to chemicals had increased during the COVID-19 pandemic due to the observed increased use of PPEs (e.g., gloves and face masks), followed by disinfection agents (66.7%, *n* = 389). Almost half of the respondents (46.5%, *n* = 274) mentioned that they felt that their exposure to chemicals in the workplace had increased. A slightly smaller proportion of participants (43.5%, *n* = 257) stated that they did not feel that due to the COVID-19 pandemic, their exposure to chemicals in their workplaces had increased. About 1-in-11 respondents felt their exposure to chemicals in their workplace (10.0%, *n* = 59), as well as their home environment (8.5%, *n* = 50), had decreased. However, respondents older than 55 years – 21.6% (*n* = 59) in the age group 55–64 years and 22.3% (*n* = 61) aged 65 and more years felt the increase of exposure to chemicals in their workplaces compared to 16.5% (*n* = 45) aged 45–54 and 14.3% (*n* = 39) in 35–44 age group. Slightly more women than men responded that they felt an increase in exposure to chemicals in their workplace – out of all respondents, who responded affirmingly, 53.3% (*n* = 146) were women and 46.7% (*n* = 128) were men.

**Table 3:** Survey questions on sources of potential exposures to chemicals during the COVID-19 pandemic, *n* (%).

**Topic of survey question regarding sources of potential exposure to chemical(s) agent(s)**	**Application of Weights**	**Has increased**	**Did not change**	**Has decreased**
Personal protective equipment	Not applied	416 (70.3%)	153 (25.8%)	23 (3.9%)
	Applied	408 (68.8%)	154 (26.0%)	31 (5.2%)
Disinfection agents	Not applied	399 (68.1%)	163 (27.8%)	24 (4.1%)
	Applied	389 (66.7%)	165 (28.3%)	29 (5.0%)
At the workplace	Not applied	270 (45.7%)	265 (44.8%)	56 (9.5%)
	Applied	274 (46.5%)	257 (43.5%)	59 (10.0%)
Household products (e.g., cleaning products, paints, arts and craft supplies)	Not applied	217 (36.8%)	352 (59.6%)	21 (3.6%)
	Applied	212 (36.2%)	349 (59.5%)	25 (4.3%)
Within the home environment (e.g., dust, indoor air)	No weights applied	182 (30.7%)	363 (61.4%)	47 (7.9%)
	Weights applied	193 (32.6%)	348 (58.9%)	50 (8.5%)
Food packaging	No weights applied	164 (27.7%)	411 (69.6%)	16 (2.7%)
	Weights applied	161 (27.2%)	421 (70.9%)	11 (1.9%)
Pharmaceuticals	No weights applied	145 (24.7%)	426 (72.7%)	15 (2.6%)
	Weights applied	146 (24.8%)	424 (72.3%)	17 (2.9%)
In the outdoor environment (e.g., soil, water, air)	No weights applied	119 (20.1%)	379 (64.0%)	94 (15.9%)
	Weights applied	141 (23.7%)	361 (60.9%)	92 (15.4%)
Food	No weights applied	92 (15.7%)	476 (81.1%)	19 (3.2%)
	Weights applied	100 (17.1%)	410 (80.4%)	15 (2.5%)
Psychoactive substances (e.g., tobacco, drugs)	No weights applied	83 (14.0%)	488 (82.6%)	20 (3.4%)
	Weights applied	78 (13.3%)	489 (83.0%)	22 (3.7%)
Personal care products (cosmetics, shampoo, shaving cream)	No weights applied	75 (12.7%)	493 (83.8%)	21 (3.5%)
	Weights applied	81 (13.9%)	487 (83.4%)	16 (2.7%)
Drinking water	No weights applied	51 (8.6%)	525 (88.9%)	15 (2.5%)
	Weights applied	52 (8.8%)	520 (88.5%)	16 (2.7%)
Non-food consumer products (e.g., textiles, shoes, sport and office items)	No weights applied	37 (6.3%)	510 (86.3%)	44 (7.4%)
	Weights applied	36 (6.2%)	508 (86.0%)	46 (7.8%)
Toys	No weights applied	27 (4.6%)	526 (90.1%)	31 (5.3%)
	Weights applied	36 (6.1%)	523 (89.6%)	25 (4.3%)
Other sources	No weights applied	134 (24.3%)	399 (72.3%)	19 (3.4%)
	Weights applied	133 (24.1%)	391 (70.6%)	29 (5.3%)

No changes due to the COVID-19 pandemic in exposure to chemicals were perceived in exposure sources like children’s toys (89.6%, *n* = 523), drinking water (88.5%, *n* = 520) and non-food consumer products (86.0%, *n* = 508). A decrease in the felt exposure to chemicals due to COVID-19 was mentioned in natural exposure sources like soil, water and air (15.4%, *n* = 92), and non-food consumer products (7.8%, *n* = 46).

### 3.2 Results of the focus group discussions

Focus group participants revealed their opinions regarding the changes in their lives and perceptions of chemical exposure resulting from the COVID-19 pandemic. Answers were categorized into (1) changes in lifestyle during the COVID-19 pandemic, (2) concerns related to the influence of the COVID-19 pandemic, and (3) positive aspects related to the influence of the COVID-19 pandemic (for details see Table 4).

**Table 4:** Categories identified during research analysis (*n* = number of persons for which theme was detected).

**Identified categories**	
**Changes in life-style during the COVID-19 pandemic**	
More often washing and disinfection of hands	*n* = 4
Place where people spend their time has changed (indoors/outdoors, including changed time proportion)	*n* = 3
Time aspect	*n* = 2
Everything has changed	*n* = 1
Market and offer have changed	*n* = 1
Life style (regime) has changed	*n* = 1
Traveling of people was less (in the context of everyday activities)	*n* = 1
There were less flights (aviation)	*n* = 1
**Concerns related to the influence of the COVID-19 pandemic**	
Excessive use of disinfecting and cleaning agents	*n* = 2
Existence of the disinfecting agents in air	*n* = 2
Use of face masks is not reasonable / stupid	*n* = 2
Changes skin of hands	*n* = 1
One type of chemicals is replaced by other types of chemicals	*n* = 1
**Positive aspects related to the influence of the COVID-19 pandemic**	
The (ambient) air has become cleaner	*n* = 2
Less diarrhoeas (due to better hand washing)	*n* = 1

Among the topics which were highlighted by several focus group participants, the use of disinfectants and cleaning agents and the time proportion spent indoors and outdoors should be mentioned. Both of them can be characterized as the main concerns raised by the focus group participants when discussing how the COVID-19 pandemic influenced human exposure to chemical substances.

“The life regime has changed, the time proportion which is spent indoors and outdoors … And with this also exposure to chemicals. Lifestyle changes…” (male, 31, higher education).

“Another, that affects a lot, are all disinfection products for surfaces, they are all around now, I would say too much. And for hands…” (male, 33, secondary education – vocational school).

“The thing that has changed in the lives of most people is the fact that they do not need to travel every day to work” (male, 31, higher education).

However, when looking at the possible health effects of the changes in the exposure to chemical substances the results were diverse. One of the focus group participants mentioned that he already experienced health effects (skin problems), and another stated that there might be long-term effects of the lifestyle changes, but the possible effects were still unknown.

“I already feel how the skin on my hands has changed during this year” (male, 33, secondary education – vocational school).

“Many [employees] work from the countryside, they telework. Maybe they are outdoors more often, but there was a moment when we were sitting only inside. It is hard to assess how this has affected us – to the positive or negative” (male, 31, higher education).

In addition, time in two different aspects was mentioned as a topic discussed by two focus group participants. One of the participants stated that lack of time was an important issue when talking about the properties of chemical substances used for disinfection. Another person mentioned that the COVID-19 pandemic had provided more time to be used for self-education in all areas, including the safety of chemicals.

“Lack of time also requires changes … choice and use of chemicals (faster evaporation, faster effect, faster results, etc.)” (female, 45, higher education).

“As for me, it seems that the COVID-19 pandemic has provided an opportunity for self-education in this [chemical safety] area” (male, 43, higher education).

## 4 Discussion

At the start of the pandemic, the available information on SARS-CoV-2 coronavirus was limited as it took time for research institutions and experts to evaluate the most effective ways of mitigating the spread of the virus. Information from the governmental institutions through articles and advertisements on social media, TV and newspapers promoted the use of personal protection, hand and surface disinfection in different facilities (schools, workplaces, also urban environments) suggesting and promoting frequent hand washing and surface disinfection (26, 27). The guidelines of the European Centre for Disease Prevention and Control on the premise cleaning and disinfection marked the different approaches of cleaning surfaces that had or had not been in contact with a potential or confirmed case of COVID-19 patient (16). However, the fear of infection caused excessive disinfection of premises that could be cleaned with neutral detergents and this resulted in added exposure to chemical products, especially in domestic settings (28).

The results of the citizen survey show that almost half of the respondents noted their increased exposure to chemicals (disinfectants) in their workplace (46.8%, *n* = 274), but a slightly smaller proportion (43.5%, *n* = 257) responded, that the exposure in their workplace had not changed. Most of the survey respondents were adult, middle-aged females with higher education and one of the first governmental restrictions was promoting remote work for all workplaces where it is possible – this could explain these similar results. When comparing the results within the group of respondents who felt their exposure to chemicals in the workplace had increased to those who did not feel the increase, no statistically significant differences between age, gender, education level and employment were found.

In the period when the citizen survey was active (September 2020 – February 2021) citizens of Latvia had already experienced the effects of the COVID-19 pandemic on their everyday life since the first lockdown had already taken place from the 12^th^ of March to the 9^th^ of June 2020 and the second emergency state was announced at the time of the survey (the 11^th^ of November 2021), lasting for almost 5 months and ending on the 1^st^ of March 2022. During the emergency states businesses were encouraged to implement remote work as much as possible. In the 3^rd^ quarter of 2020, 70,500 people were working remotely and it doubled to 167,600 workers in the first quarter of 2021 (29). This shift could explain the results where almost half of the respondents felt that the exposure to chemicals in their workplace had increased. However, supportive data from our research are not available as telework was not addressed in our questionnaire.

It is important to mention that about 2-in-3 citizen survey respondents (66.7%, *n* = 389) reported that they felt that disinfecting agents had increased their exposure to chemical substances due to the COVID-19 pandemic. Typically, skin, respiratory and gastrointestinal tract sensibilizations were the most commonly recognized health effects of excessive use of disinfectants (30). One of the focus group participants in our study reported having adverse skin health effects due to frequent washing and disinfection of hands. Four others admitted that during the COVID-19 pandemic, they had experienced more frequent hand washing and sanitizing. In addition, the opinions that the use of cleaning and disinfectant agents was excessive and the disinfectants stayed in the air were also expressed. Thus, the results from our study support the concerns in Europe, that the improper and excessive use of disinfectants and cleaning products can lead to different health effects, including poisonings both in adult and children’s populations (26–28).

Although the increased amount of waste is a well-described effect of the pandemic (2, 8, 11, 31), none of the data of our research shows an increased concern on this topic. Even though most of the respondents acknowledged that due to the use of PPEs, they felt that their exposure to chemicals had increased (68.8%, *n* = 408), they had not recognized the circular economy aspect of PPE being mostly single-usable and ending up as trash, therefore causing a waste management problem. This indicates the lack of overall knowledge on recycling and waste management and the need for better public education on these topics. Infrastructure and accessibility (proximity) of recycling containers is one of the reasons for not recycling (18), however, governmental actions are built toward promoting recycling by adapting a deposit system for glass, metal and polyethylene terephthalate (PET) containers (32) and by slowly promoting circular economy and continuing to establish local waste management facilities (33). The data for 2020 shows that households generated the most share amount of waste in Latvia (22.6% share of total waste), which was more than double of the EU average (9.4% share of total waste) (34). This indicates that further work and research should be promoted in this area to minimize the amount of waste produced by households. Another proactive activity to minimize household-generated waste would be to raise public awareness of this issue, especially among children, since the impact is not only individual but can also spell over and influence the habits and knowledge of family members – a suggestion brought up by focus group participants in another research paper based on the results of the HBM4EU citizen survey (23).

Healthcare waste is not separated from other categories and labeled as “hazardous waste.” No public data is available on the amount of waste generated in Latvia during the COVID-19 pandemic, but presumably, the amount has increased similar to other European countries, resulting in tonnes of “hazardous waste.” Furthermore, some waste recycling facilities use manual labor to sort domestic waste and the fear of sorting possibly infectious waste has impeded the domestic waste recycling process and the amount of unrecycled domestic waste has increased (35).

According to the published data, decisions of the national governments that restricted most of the everyday activities during the COVID-19 pandemic (e.g., lockdown), caused a drop in air pollution (36). This was also the topic addressed in our focus group discussions. In general, less aviation, less use of vehicles resulting from a stay-at-home policy, decrease in heating due to the closure of workplaces (e.g., offices because of the telework or sites because of quarantine), non-functioning of industries, etc. have been reported as major explanations (2, 37). When looking at the topics mentioned in our focus group discussions, only less travel (both aviation and road transport) had been discussed. In the citizen survey, 15.4% (*n* = 92) mentioned they felt that their exposure to chemicals in the environment (soil, water, air) had decreased. Our study results on this topic support the results of studies carried out previously (23).

The lifestyle changes caused by the COVID-19 pandemic toward spending more time indoors were mentioned by several (*n* = 3) focus group participants. These changes included not having to travel to work and working from home, resulting in changes in the proportion of time spent indoors vs. time spent outdoors (an increase in the amount of time spent indoors). Furthermore, not only the time spent indoors but also an increase in the exposure to chemical substances in their home environment had been reported in our study by approximately one-third of the citizen survey participants (32.6%, *n* = 193). It has been already well described that the sources of indoor pollutants include furniture, utility, building materials, the presence of occupants and their activities, in particular, burning gas and other fossil fuels for cooking and heating, tobacco smoking, cleaning with detergents and personal care products, burning candles or incense (38). In addition to the increased use of disinfecting agents (17), some of the mentioned activities were more often performed during the governmental restrictions to mitigate the spreading of the SARS-CoV-2 virus were implemented, e.g., cooking and heating. Health effects resulting from indoor pollution during the COVID-19 pandemic were less described if compared to reduced daily physical activity and increased sedentary time during the COVID-19 pandemic which was also related to the increased time spent indoors (39).

Citizen survey results show that respondents felt that the most decrease in chemical substance exposure during the COVID-19 pandemic was in the environment (soil, water, air). Some part of this decrease could be due to the decreased activity of different industries and factories, however, this was not mentioned in focus group discussions. Such findings might be explained by the fact that the number of COVID-19 cases during the 1st year of the pandemic was rather low and the Latvian government did not have to announce a full lockdown, therefore there was no major effect on the work of the industrial sites.

No specific groups could be distinguished to whom the COVID-19 pandemic had urged to increase their knowledge of chemical exposure. Data from the literature review shows that positive examples from society are important for making environmentally and human health-friendly choices. A positive influence can be promoted via social media, including collaboration with public figures (influencers, TV stars) (18, 23, 40). Environmental education at an early age is also mentioned as a way to raise public awareness on the topic of chemical pollution and waste (23, 40). A targeted approach where specifically vulnerable groups are identified and addressed could give better results since a tailored approach is more likely to reach the desired part of the community (41).

Several limitations have been identified for our study. The use of the online survey as a method to gather survey data may exclude some of the groups of respondents from the sample by default (e.g., persons with low education and digital literacy, people living in remote areas, older adult, etc.). Another aspect is the use of Latvian as the language of the survey. Even though it is the only national language, the Russian-speaking community might have participated at a lower response rate. One more limitation is related to the use of a non-probability sampling method which allows quickly gathering information from participants. This has resulted in a sample that is not representative of the demographic profile of the Latvian population. In addition, the number of respondents participating in this survey was slightly smaller than the desired sample size. To best overcome this limitation, we used weights based on age and gender. We were not able to weight data in terms of education or work experience as such population estimates were not available from the Central Statistical Bureau of Latvia for the study period. The unequal distribution of participants with different educational levels was also a major problem for the focus group discussions. Therefore, the results of the survey and focus groups carried out in Latvia more reflect the opinion of higher educated inhabitants. At the same time, the level of awareness even of those individuals is very diverse (from knowing nothing to specifying chemicals already being addressed through Human Biomonitoring), and that means that the awareness level of chemical exposure is not linked with low education level.

Despite all of these limitations, we believe that the results of our study provide descriptive and useful information about the changes in perception and concerns on chemical substances during the COVID-19 pandemic.

## 5 Conclusion

According to the quantitative and the qualitative data of our research, the excessive use of disinfectant products has been the main concern of citizens in Latvia regarding chemical exposure resulting from the COVID-19 pandemic. The use of these products is a source of indoor air pollution and has an impact on human health due to primary and secondary emissions. It seems that the COVID-19 pandemic has not only increased the use of chemical products in Latvia but also promoted an interest in the safe and healthy use of chemicals which could be useful to raise the awareness of the general public. Further research should be carried out to distinguish groups where the knowledge of chemical exposure is especially low. For these vulnerable groups, a specifically tailored approach rather than an overall educational campaign should be used to gain the best results in raising awareness regarding this topic.

## Data availability statement

Data sharing of focus group discussions is not applicable as the data consist of focus group transcripts and cannot be shared due to confidentiality. The raw data from the HBM4EU citizen survey supporting the conclusions will be made available by authors, without undue reservation.

## Ethics statement

The studies involving humans were approved by the Ethics Committee of Rīga Stradiņš University (protocol No. 22–2/250/2021, 14 April 2021). The studies were conducted in accordance with the local legislation and institutional requirements. The participants provided their written informed consent to participate in this study.

## Author contributions

LA: Writing – review & editing, Writing – original draft, Investigation, Conceptualization. LP: Writing – review & editing, Visualization, Validation, Software, Methodology, Formal analysis, Data curation. LM: Writing – review & editing, Writing – original draft, Supervision, Methodology, Data curation. IM: Writing – review & editing, Supervision, Project administration, Methodology, Funding acquisition. IV: Writing – review & editing, Supervision, Resources, Project administration, Funding acquisition. LK: Writing – review & editing.

## Footnotes

^1^ www.stradavesels.lv

^2^ www.stradavesels.lv
